# New Insights into the Steen Solution Properties: Breakthrough in Antioxidant Effects via NOX2 Downregulation

**DOI:** 10.1155/2014/242180

**Published:** 2014-04-16

**Authors:** Roberto Carnevale, Giuseppe Biondi-Zoccai, Mariangela Peruzzi, Elena De Falco, Isotta Chimenti, Federico Venuta, Marco Anile, Daniele Diso, Elena Cavarretta, Antonino G. M. Marullo, Patrizio Sartini, Pasquale Pignatelli, Francesco Violi, Giacomo Frati

**Affiliations:** ^1^Department of Internal Medicine and Medical Specialties, Sapienza University of Rome, 00161 Rome (RM), Italy; ^2^Department of Medico-Surgical Sciences and Biotechnologies, Sapienza University of Rome, 04100 Latina (LT), Italy; ^3^Department of Thoracic Surgery, Sapienza University of Rome, 00161 Rome (RM), Italy; ^4^Fondazione Eleonora Lorillard Spencer Cenci, 00185 Rome (RM), Italy; ^5^Department of Heart and Great Vessels, Sapienza University of Rome, 00161 Rome (RM), Italy; ^6^Department of AngioCardioNeurology, IRCCS NeuroMed, 86077 Pozzilli (IS), Italy

## Abstract

Ex vivo lung perfusion (EVLP) allows perfusion and reconditioning of retrieved lungs for organ transplantation. The Steen solution is specifically designed for this procedure but the mechanism through which it elicits its activity is still to be fully clarified. We speculated that Steen solution may encompass antioxidant properties allowing a reestablishment of pulmonary tissue homeostasis. Blood samples from 10 healthy volunteers were recruited. Platelets and white cells were incubated with Steen solution or buffer solution as control and stimulated with suitable agonists. Reactive oxidant species (ROS), soluble NOX2 (sNOX2-derived peptide), a marker of NADPH oxidase activation, p47^phox^ translocation to cell membrane and isoprostanes production, as marker of oxidative stress, and nitric oxide (NO), a powerful vasodilator and antioxidant molecule, were measured upon cell stimulation. The Steen solution significantly inhibited p47^phox^ translocation and NOX2 activation in platelets and white cells. Consistent with this finding was the reduction of oxidative stress as documented by a significantly lowered formation of ROS and isoprostanes by both platelets and white cells. Finally, cell incubation with Steen solution resulted in enhanced generation of NO. Herewith, we provide the first evidence that Steen solution possesses antioxidant properties via downregulation of NADPH oxidase activity and enhanced production of NO.

## 1. Introduction

Organ transplantation improves quality and expectancy of life of patients with end-stage organ failure. The claim for transplantation is expected to increase because of the prolonged expectancy of life. However, despite an exponential growth in the 90s, the absolute number of donors and their rate per million population (pmp) did not further improve [1, 2]. To overcome these hurdles and increase the pool of organs, national health systems along with health professionals directly involved in this field contributed to producing policies aimed at optimizing all phases of the process of organ donation and transplantation after brain death. However, this strategy, despite its proved efficacy, has partially failed to balance the organ offer to its growing demand [3].

In this context, lung transplantation (LT) is considered a viable therapeutic option for a selected group of patients with end-stage lung disease. However, the increasing number of patients on the waiting list clearly exceeds the number of available donors; this is because approximately only an average of 20% of the potential multiorgan donors is currently used for LT [4]. This contributes to an increase in waiting time and mortality on the waiting list that often exceeds 20% [5]. Most of the reasons why the wide majority of the donor lungs are considered unsuitable for transplantation are related to lung injury occurring during trauma or after brain death and to the complications associated with a prolonged intensive care unit (ICU) stay. Consequently, a number of strategies have been proposed: the use of marginal donors [6], living related LT [7, 8], or non-heart beating donation [9]. Notwithstanding these improvements, the number of transplants has hitherto not significantly increased. Behind these assertions, the concept of lung procurement from marginal and extended donors and the possibility of “organ reconditioning” have been successfully used for transplantation and this new concept is gaining large acceptance.

Normothermic ex vivo lung perfusion (EVLP) allows perfusion of the retrieved lungs in an ex vivo circuit with functional reassessment and reconditioning before transplantation, irrespective of the protocol adopted: the Lund model or the Toronto model [9–11]. This novel technique utilizes an original priming/perfusion solution specifically designed for this procedure such as the Steen solution. In the Lund model, the EVLP procedure is performed with a mixture of Steen solution and washed erythrocytes in order to reach a hematocrit of 15%; besides, the left atrium is kept open, offering the possibility of a normal cardiac output (5-6 L/min) during EVLP. In the Toronto protocol, the EVLP procedure is carried out with an “acellular” Steen solution and a closed left atrium achieving thus only 40% of the normal cardiac output. Irrespective of the protocol adopted, the reason why this technique seems to work so profitably is mainly correlated to different variables: the use of steroids and high dose of antibiotics, the high osmotic power of the solution, and the effects of the leukocyte depletion filter interposed on the circuit. The EVLP procedure is a typical model of ischemia reperfusion which is associated with reactive oxidant species (ROS) formation; such a phenomenon may theoretically preclude optimal lung perfusion and consequently the most favorable clinical outcome as ROS has negative effect on endothelial function via inhibition of nitric oxide (NO) activity. We speculated that Steen solution may possess antioxidant properties which may blunt ROS formation thus improving lung perfusion. To address this issue, we explored an ex vivo experimental model consisting in challenging the Steen solution with blood cells activated to produce ROS generated by NOX2, the catalytic subunit of NADPH oxidase.

## 2. Material and Methods

### 2.1. Subjects

We studied ten healthy volunteers (5 males, 5 females, age: 33.5 ± 5.7) (Table 1). Written informed consent was obtained from all subjects. The study was approved by the local ethical committee (December 12, 2013, Protocol number 3010) and was conducted in accordance with the principles embodied in the Declaration of Helsinki.

Blood samples were obtained after a 12-hour fast between 8.00 and 9.00 a.m. from subjects undergoing routine biochemical analysis including total cholesterol and glucose. Samples, obtained from healthy subjects after supine rest for at least 10 min, were taken into tubes with 104 3.8% sodium citrate or EDTA.

### 2.2. Laboratory Analyses

All materials were from Sigma-Aldrich (Milan, Italy) unless otherwise specified. The Steen solution was purchased from Vitrolife (Göteborg, Sweden). It is a physiological salt solution containing human serum albumin (providing normal oncotic pressure in order to prevent edema formation), dextran (a mild scavenger which coats and protects endothelium from subsequent excessive leucocyte interaction and thrombogenesis), and characterized by a prevalent extracellular electrolyte composition (low potassium, decreasing free radical generation and preventing vascular spasm under normothermic conditions).

### 2.3. Platelet Preparation and Activation

To acquire platelet-rich plasma (PRP), samples were centrifuged for 15 minutes at 180 g. In order to avoid leukocyte contamination, only the top 75% of the PRP was collected according to Pignatelli et al. [12]. Platelet pellets (PLT) were obtained by double centrifugation (5 minutes, 300 g) of PRP. Acid/citrate/dextrose (ACD) (1 : 7 v/v) was added to avoid platelet activation during processing. Samples were suspended in HEPES buffer (buffer solution, BS) in presence of 0.1% albumin, pH 7.354 (2 × 10^5^ PLT/mL, unless otherwise noted), or in presence of 1 mL of Steen solution (2 × 10^5^ PLT/mL, unless otherwise noted) and stimulated with or without 0.5 mM arachidonic acid (AA) in presence or absence of the inhibitor of NADPH oxidase (apocynin, 50 *μ*M) at room temperature for 15 minutes. Cells were separated from the supernatant by centrifugation (5 minutes, 300 g) and the two fractions, cells and supernatants, were stored at −80°C until analysis.

### 2.4. Human Polymorphonuclear Leukocyte Preparation and Activation

Polymorphonuclear leukocytes (PMNs) were isolated from freshly taken EDTA blood from healthy volunteers by dextran enhanced sedimentation of red blood cells, Ficoll-Histopaque density centrifugation, lysis of remaining erythrocytes with distilled water, and washing of cells with Hank's balanced salt solution (HBSS) (buffer solution, BS) in absence of any divalent cations. Finally, the cell pellet was suspended in 1 mL of HBSS or 1 mL of Steen solution at the final concentration of 1 × 10^6^ cells/mL and stimulated with phorbol 12-myristate 13-acetate (PMA) (10 *μ*M) in presence or absence of the inhibitor of NADPH oxidase (apocynin, 50 *μ*M) at room temperature for 15 minutes. Cells were separated from the supernatant by centrifugation (5 minutes, 300 g) and stored at −80°C until analysis.

### 2.5. Lymphocytes/Monocytes Preparation

Blood samples were collected in heparinized tubes (10 IU/mL). Lymphocytes/monocytes (LYM/MON) (1 × 10^6^ cells/mL) were isolated after centrifugation of the blood from healthy volunteers (*n* = 5, healthy subjects) with a polysucrose-sodium diatrizoate solution, 1.077 g/mL density, and 280 mOsm osmolarity (Lymphoprep; Nycomed, Oslo, Norway) at 800 g at 20°C. The LYM/MON cell layer was collected and the cells were thus washed two times in a solution of cold phosphate-buffered saline (pH 7.2) (PBS), supplemented with 1% fetal calf serum and 2 mmol/L EDTA (Sigma-Aldrich). Finally, the cell pellet was suspended in 1 mL of PBS (buffer solution, BS) or 1 mL of Steen solution at the final concentration of 1 × 10^6^ cells/mL. The cell suspension was stimulated with or without lipopolysaccharide (50 pg/mL) (LPS) in presence or absence of the inhibitor of NADPH oxidase (apocynin, 50 *μ*M) at room temperature for 15 minutes. Cells were separated from the supernatant by centrifugation (5 minutes, 300 g) and stored at −80°C until analysis.

### 2.6. Assessment of Reactive Oxygen Species (ROS) Production by Flow Cytometry

Cells* suspensions* were incubated with 2′,7′-dichlorofluorescin diacetate (5 *μ*M) (15 minutes at 37°C). 10 *μ*L of each activated and unactivated sample was diluted with 1 mL of buffer solutions or Steen solution and analyzed by flow cytometry. ROS production was expressed as mean fluorescence (MF).

### 2.7. Analysis of sNOX2-dp and 8-iso-PGF2*α*-III

Extracellular levels of soluble NOX2-derived peptide (sNOX2-dp), a marker of NADPH oxidase activation, were detected by ELISA method as previously described by Pignatelli et al. [12]. The peptide was recognized by the specific monoclonal antibody against the amino acidic sequence (224–268) of the extra membrane portion of NOX2 (catalytic core of NADPH oxidase), which was released in the medium upon platelet activation. Values were expressed as pg/mL; intra-assay and interassay coefficients of variation were 5.2% and 6%, respectively. Cells 8-iso-PGF2*α*-III production was measured by EIA assay method (Tema Ricerca, Italy) and expressed as pmol/L. Intra- and interassay coefficients of variation were 5.8% and 5.0%, respectively.

### 2.8. Cells NOx Measurement

A colorimetric assay kit (Tema Ricerca, Italy) was used to determine the nitric oxide metabolites nitrite and nitrate (NOx) in the supernatant of platelet and white cells in presence or absence of the Steen solution (1 mL). Intra-assay and interassay coefficients of variation were 2.9% and 1.7%, respectively.

### 2.9. Membrane and Cytoplasmic Proteins Extraction

To analyze the specificity of Steen solution in blocking cells NADPH oxidase activation, the effect of this solution was analyzed on the translocation of p47^phox^ from cytosol to membranes in agonists-activated cells according to Fortuño et al. [13].

Briefly, the extraction of membrane and cytoplasmic proteins was performed by using the ProteoJET Membrane Protein Extraction Kit (Fermentas International Inc., Maryland, USA) [13].

### 2.10. Western Blot Analysis of p47^phox^

Activated and unactivated samples were suspended in a 2X lysis buffer (5 mM EDTA, 0.15 mol NaCl, 0.1 mol Tris, pH 8.0, and 1% triton and protease inhibitor cocktail). Equal amounts of protein (30 *μ*g/lane) estimated by Bradford assay were solubilized in a 2X Laemmli buffer containing 2-mercaptoethanol and loaded in a denaturing SDS/10% polyacrylamide gel. Western blot analysis was performed with monoclonal anti-p47^phox^ (1 *μ*g/mL) incubated overnight at 4°C. Immune complexes were detected by enhanced chemiluminescence. The developed spots were calculated by densitometric analysis on a NIH image 1.62f analyzer and the value was expressed in arbitrary unit (AU).

### 2.11. Statistical Analysis

Continuous variables are reported as mean ± standard deviation and categorical variables as *n* (%). Continuous variables were compared with Student's *t*-test and ANOVA, as appropriate. Statistical significance was set at the 0.05 2-tailed level, with *P* values unadjusted for multiplicity unreported throughout. Computations were performed with SPSS 20 (IBM, Armonk, NY, USA).

### 2.12. Sample Size

We computed the minimum sample size with respect to a two-tailed one-sample paired Student's *t*-test, considering the following: (i) that |*δ* | ≥8 pg/mL would be a clinically relevant difference in NOX2 levels between stimulated cells treated without or with Steen solution, (ii) a 5 pg/mL standard deviation (SD) for the paired difference; (iii) type-I error probability *α* = 0.05 and power 1 − *β* = 0.90. These assumptions lead to *n* = 9.

## 3. Results

### 3.1. Role of the Steen Solution in ROS Production and 8-iso-PGF2*α*-III Production

The production of ROS in PLT, PMNs, and LYM/MON was evaluated by flow cytometric analysis using the fluorescent probe 2′,7′-diclorofloresceinaacetate (DCFH-DA). As a positive control of ROS inhibition, we repeated the experiments with apocynin, the specific inhibitor of NADPH oxidase.

**Figure 1:**
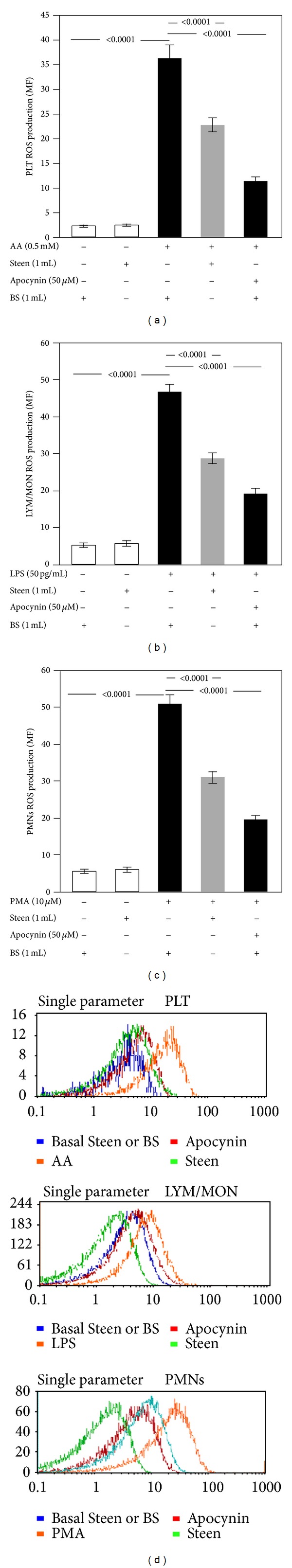
Role of Steen solution in the cellular production of ROS. In vitro study: ROS production in cells suspended in the buffer solution (BS) or Steen solution and stimulated with or without arachidonic acid (AA) (0.5 mM) for platelet (PLT) (a), lipopolysaccharide (LPS) (50 pg/mL) for lymphocytes/monocytes (LYM/MON) (b), and phorbol 12-myristate 13-acetate (PMA) (10 *μ*M) for polymorphonuclear leukocytes (PMNs) (c). Cells were treated or not with apocynin (50 *μ*M). Experiments were led on 10 subjects. White bars in the histogram graph represent control. A representative cytometry analysis of reactive oxygen species (ROS) production (d).

As shown in Figures 1(a), 1(b), 1(c), and 1(d), we observed an increased ROS production in cells suspended in the buffer solution and stimulated with AA (0.5 mM) for PLT, LPS (50 pg/mL) for LYM/MON, and PMA (10 *μ*M) for PMNs compared to nonstimulated cells. In presence of Steen solution (1 mL), we observed a reduction of ROS production in PLT (22.6 ± 4.1 MF versus 36.2 ± 8.0 MF; *P* < 0.0001), LYM/MON (28.6 ± 4.5 MF versus 46.6 ± 6.0 MF; *P* < 0.0001), and PMNs (31.0 ± 4.7 MF versus 50.8 ± 7.1 MF; *P* < 0.0001) compared to stimulated cells in buffer solutions. The positive control, apocynin, significantly inhibited the production of ROS (Figures 1(a), 1(b), and 1(c)) (*P* < 0.0001).

**Figure 2:**
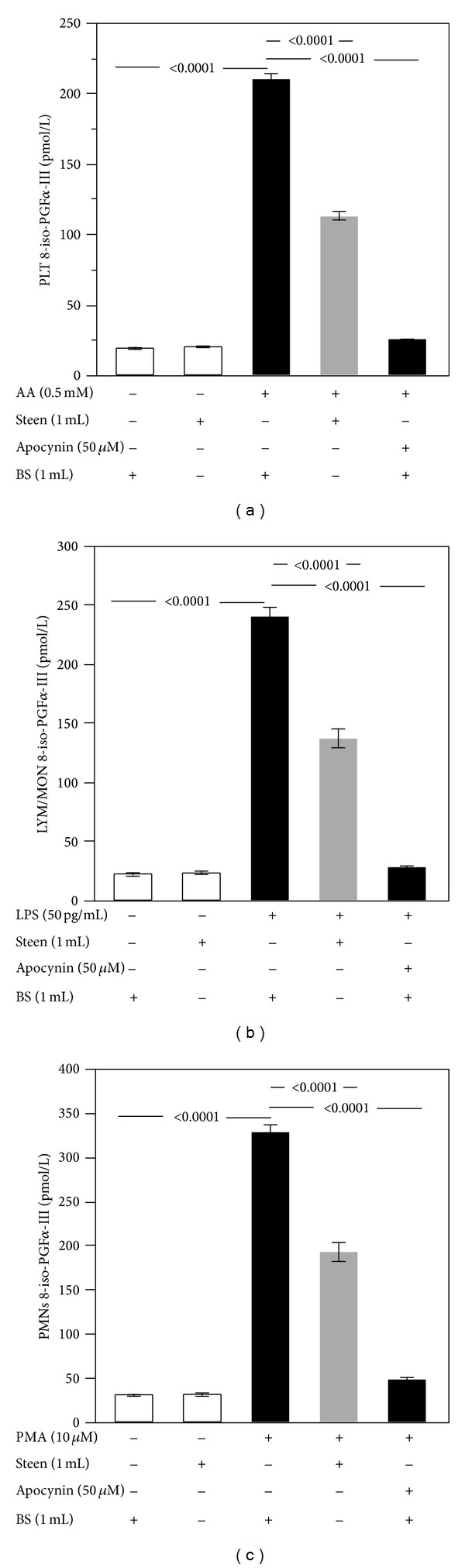
Role of Steen solution in the cellular production of 8-iso-PGF2*α*-III. In vitro study: 8-iso-PGF2*α*-III production in cells suspended in the buffer solution (BS) or Steen solution and stimulated with or without arachidonic acid (AA) (0.5 mM) for platelet (PLT) (a), lipopolysaccharide (LPS) (50 pg/mL) for lymphocytes/monocytes (LYM/MON) (b), and phorbol 12-myristate 13-acetate (PMA) (10 *μ*M) for polymorphonuclear leukocytes (PMNs) (c). Cells were treated or not with apocynin (50 *μ*M). Experiments were led on 10 subjects. White bars represent control.

Coincidentally, with enhanced ROS formation, 8-iso-PGF2*α*-III production increased in cells suspended in buffer solutions and stimulated with AA (0.5 mM) for PLT, LPS (50 pg/mL) for LYM/MON, and PMA (10 *μ*M) for PMNs compared to nonstimulated cells (Figures 2(a), 2(b), and 2(c)). Even in this case the Steen solution (1 mL) was able to reduce 8-iso-PGF2*α*-III production in PLT (112.3 ± 9.1 pmol/L versus 209.7 ± 10.3 pmol/ L; *P* < 0.0001), LYM/MON (136.6 ± 24.4 pmol/L versus 239.9 ± 24.1 pmol/L; *P* < 0.0001), and PMNs (192.2 ± 33.1 pmol/L versus 327.6 ± 25.1 pmol/L; *P* < 0.0001) (Figures 2(a), 2(b), and 2(c)) compared to stimulated cells in buffer solutions. The positive control, apocynin, significantly inhibited the production of 8-iso-PGF2*α*-III (Figures 2(a), 2(b), and 2(c)) (*P* < 0.0001).

### 3.2. Effect of the Steen Solution on NADPH Oxidase

To analyze the pathway involved in oxidative stress inhibition by the Steen solution, we studied the activation of NADPH oxidase by measuring the extra membrane portion of NOX2 (the catalytic core of NADPH oxidase), which is released in the medium upon cell activation, and p47^phox^ expression on cell membrane, a key subunit for NADPH oxidase activation [14].

**Figure 3:**
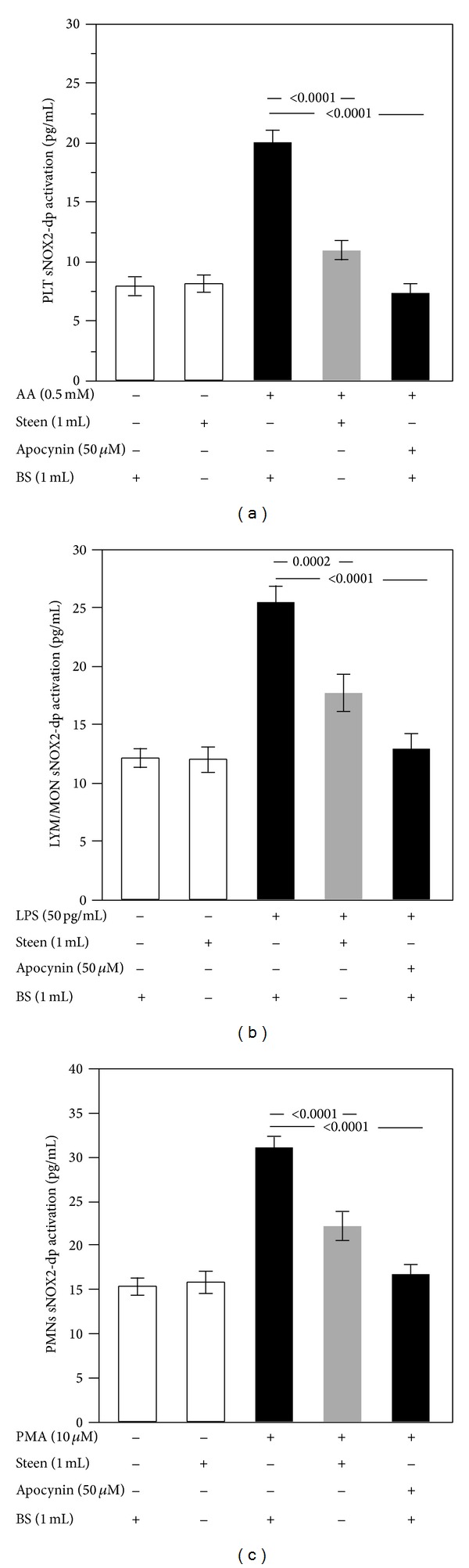
Role of Steen solution in the cellular NADPH oxidase activation. In vitro study: NADPH oxidase activation, evaluated by the release of sNOX2-dp, in cells suspended in the buffer solution (BS) or Steen solution and stimulated with or without arachidonic acid (AA) (0.5 mM) for platelet (PLT) (a), lipopolysaccharide (LPS) (50 pg/mL) for lymphocytes/monocytes (LYM/MON) (b), and phorbol 12-myristate 13-acetate (PMA) (10 *μ*M) for polymorphonuclear leukocytes (PMNs) (c). Cells were treated or not with apocynin (50 *μ*M). White bars represent control.

As shown in Figures 3(a), 3(b), and 3(c), Steen solution significantly decreased the activation of NADPH oxidase in each cell type (10.8 ± 2.4 pg/mL versus 20.1 ± 3.1 pg/mL in PLT, *P* < 0.0001; 17.6 ± 4.7 pg/mL versus 25.4 ± 4.3 pg/mL in LYM/MON, *P* = 0.0002; and 22.1 ± 4.7 pg/mL versus 31.1 ± 3.5 pg/mL in PMNs, *P* < 0.0001) as observed by reduced levels of sNOX2-dp, compared to stimulated cells in buffer solutions. The positive control, apocynin, significantly inhibited the activation of NADPH oxidase by reducing the levels of sNOX2-dp (Figures 3(a), 3(b), and 3(c)) (*P* < 0.0001).

**Figure 4:**
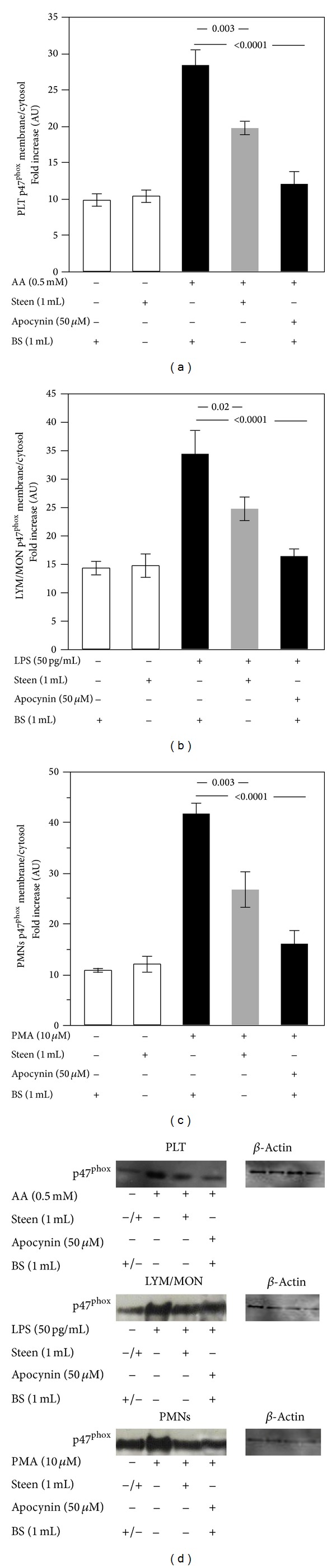
Role of Steen solution in the cellular p47^phox^ translocation. In vitro study: p47^phox^ translocation in cells suspended in the buffer solution (BS) or Steen solution and stimulated with or without arachidonic acid (AA) (0.5 mM) for platelet (PLT) (a), lipopolysaccharide (LPS) (50 pg/mL) for lymphocytes/monocytes (LYM/MON) (b), and phorbol 12-myristate 13-acetate (PMA) (10 *μ*M) for polymorphonuclear leukocytes (PMNs) (c). Cells were treated or not with apocynin (50 *μ*M). A representative Western blot analysis of membrane p47^phox^ (d). White bars represent control.

Concerning the translocation of p47^phox^ on cell membrane, we observed that Steen solution inhibited the translocation of this subunit of NADPH oxidase in all cell types (19.6 ± 1.5 AU versus 28.3 ± 3.5 AU in PLT, *P* = 0.003; 24.6 ± 3.5 AU versus 34.3 ± 7.1 AU in LYM/MON, *P* = 0.02; and 26.6 ± 6.1 AU versus 41.6 ± 3.5 AU in PMNs, *P* = 0.003) compared to stimulated cells in buffer solutions (Figures 4(a), 4(b), 4(c), and 4(d)). The positive control, apocynin, significantly inhibited the expression of p47^phox^ (Figures 4(a), 4(b), 4(c), and 4(d)) (*P* < 0.0001).

**Figure 5:**
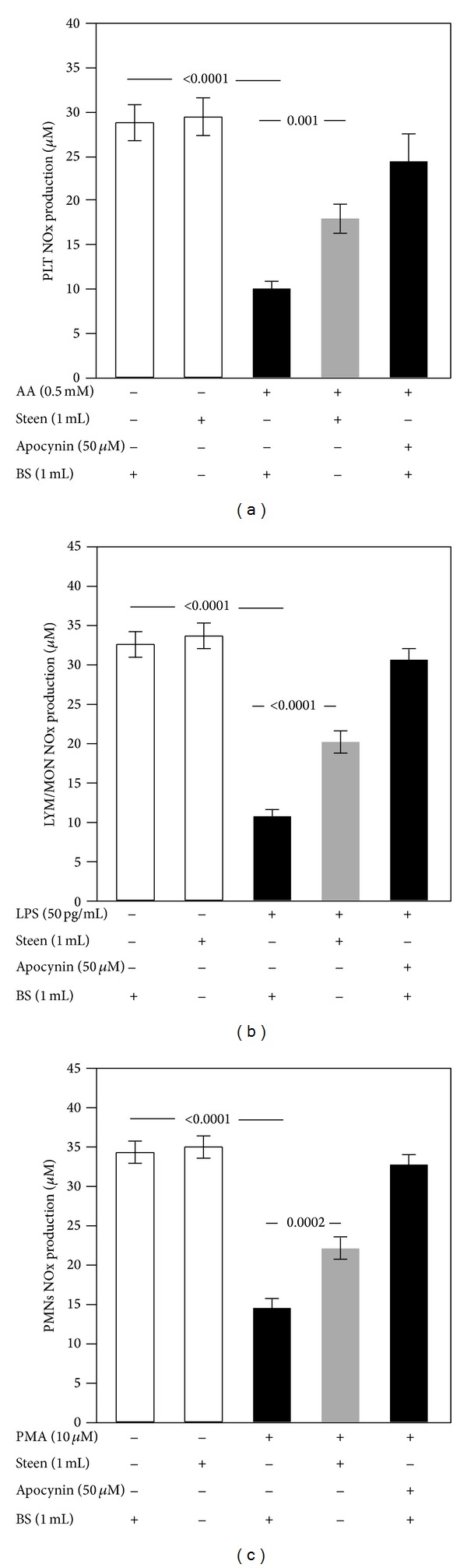
Role of Steen solution in the cell nitric oxide biodisponibility. In vitro study: nitric oxide biodisponibility, evaluated by the production of nitrite/nitrate (NOx), in cells suspended in the buffer solution (BS) or Steen solution and stimulated with or without arachidonic acid (AA) (0.5 mM) for platelet (PLT) (a), lipopolysaccharide (LPS) (50 pg/mL) for lymphocytes/monocytes (LYM/MON) (b), and phorbol 12-myristate 13-acetate (PMA) (10 *μ*M) for polymorphonuclear leukocytes (PMNs) (c). Cells were treated or not with apocynin (50 *μ*M). White bars represent control.

Cells suspended in buffer solution and incubated with the specific agonists significantly reduced NO generation compared with nonactivated cells (10.1 ± 2.5 versus 29.3 ± 6.4 *μ*M in PLT, *P* < 0.0001, Figure 5(a); 10.6 ± 2.7 versus 33.5 ± 4.9 *μ*M in LYM/MON, *P* < 0.0001, Figure 5(b); and 14.4 ± 3.4 versus 34.8 ± 4.1 *μ*M in PMNs, *P* < 0.0001, Figure 5(c)). Cell incubation with Steen solution enhanced NO generation (+44% for PLT, *P* = 0.001; +47% for LYM/MON, *P* < 0.0001; and +35% for PMNs, *P* = 0.0002) compared to stimulated cells in buffer solutions. Inhibition of oxidative stress by apocynin elicited NO overgeneration by activated cells (Figures 5(a), 5(b), and 5(c)).

## 4. Discussion

We report for the first time that the Steen solution reveals antioxidant properties by downregulating ROS-derived NOX2 activation.

ROS are chemically unstable molecules which rapidly react with other molecules giving rise to oxidized products such as oxidized LDL, peroxynitrite, or protein adducts [15]. At physiologic concentration, ROS serve as second messengers and, accordingly, they behave as intracellular signals for cell activation. Among the cell types in which ROS act as second messengers, platelets represent a typical example of ROS involvement in the process of activation. Thus, upon activation by common agonists, platelets produce several types of ROS such as superoxide anion or hydrogen peroxide that in turn contribute to propagation of platelet aggregation [16]. There are several enzymes that, upon activation, produce ROS, including myeloperoxidase, NADPH oxidase, xanthine-oxidase, or uncoupled eNOS. Among them, however, NADPH oxidase is the most important cell producer of ROS. Platelets possess all the subunits of the NADPH oxidase including gp91phox (NOX2) that is its catalytic subunit [17–19]. Activation of platelet NADPH oxidase is crucial for O_2_^−^ production as shown by its complete suppression in case of NADPH oxidase hereditary deficiency. Regarding this, we have shown that platelets from patients with chronic granulomatous disease (X-CGD) have an almost complete suppression of platelet O_2_^−^ production as a consequence of the hereditary deficiency of NOX2 [18]. Using blood cells as a tool to explore if the Steen solution has antioxidant properties, we found that two markers of oxidative stress, namely, ROS and isoprostane formation, were reduced upon cell incubation with the solution. Further support to the antioxidant property of the Steen solution was provided by demonstrating an increase of NO in the supernatant of stimulated cells incubated with the Steen solution. Thus, NO is a powerful vasodilator and antiaggregating molecule and is rapidly inactivated by ROS, which ultimately determine impaired NO generation.

Upon activation, blood cells release F2-isoprostanes, in particular 8-iso-PGF2*α*, a chemically stable compound derived from nonenzymatic oxidation of arachidonic acid (AA) [19]. We recently demonstrated that formation of platelet isoprostanes depends upon NADPH oxidase activation and contributes to the process of platelet recruitment via activation of the GpIIb/IIIa [17]. Thus, the reduction of isoprostanes yielded by the Steen solution leads us to hypothesize that such effect could be related to NADPH oxidase downregulation. Accordingly, we found that Steen solution inhibited NOX2 activation as shown by the significant lowering of sNOX2-dp in the supernatant of stimulated cells incubated with the Steen solution. This finding was corroborated by experiments through which we could demonstrate that the Steen solution impaired the translocation on platelet membrane of the cytosolic subunit p47^phox^, which has a crucial role in the assembly process of NADPH subunits and is, therefore, essential for NOX2 activation. Taken together, these findings suggest that, in blood cells, the Steen solution could act as an antioxidant via downregulation of NOX2. Although we do not have the data, we can speculate that Steen solution will also act on erythrocytes. We cannot exclude that other enzymatic pathways may be implicated in the impaired ROS production elicited by the Steen solution. Furthermore, the exact mechanism through which the Steen solution downregulates NOX2 is an open issue that deserves further investigation. Finally, it is arguable that the Steen solution could act as antioxidant also at endothelial level, but this hypothesis must be further explored.

In conclusion, our data suggests that the Steen solution plays an important antioxidant role by impairing the formation of ROS-derived NOX2 activation in several cell types. This finding provides new insights into the mechanism through which the Steen solution allows lung reconditioning during EVLP.

Further in vivo studies are currently ongoing and will shed additional lights on the translational value of the approach hereby proposed.

## Figures and Tables

**Table 1:** Characteristics of healthy subjects.

Patients	*N* = 10
Age (years)	32.8 ± 3.1
Males (%)	5 (50)
Body mass index (kg/m^2^)	21.1 ± 4.5
Systolic blood pressure (mmHg)	126 ± 11
Diastolic blood pressure (mmHg)	79 ± 10
Total cholesterol (mg/dL)	184 ± 7
Low density lipoprotein cholesterol (mg/dL)	97 ± 10
Fasting glycemia (mg/dL)	85 ± 10
Smokers	No
Gas exchange assessed by spirometry test	Within normal ranges
